# Obstacles to access to community care in urban senior-only households: a qualitative study

**DOI:** 10.1186/s12877-022-02816-y

**Published:** 2022-02-14

**Authors:** Ni Gong, Ya Meng, Qin Hu, Qianqian Du, Xiaoyu Wu, Wenjie Zou, Mengyao Zhu, Jiayan Chen, Lan Luo, Yu Cheng, Meifen Zhang

**Affiliations:** 1 School of Nursing, Jinan University, Guangzhou, Guangdong China; 2 School of Nursing, Sun Yat-sen University, 74 Zhongshan Road 2, Guangzhou, 510080 Guangdong China; 3 School of Sociology and Anthropology, Sun Yat-sen University, Xingang West Road, Haizhu District, Guangzhou, 510399 Guangdong China; 4 Qizhi Social Work Service Center, Tianhe District, Guangzhou, Guangdong China; 5 Hongshan Street Community Health Service Center, Huangpu District, Guangzhou, Guangdong China

**Keywords:** Older adults, Community care, Utilization, Accessibility, Obstacle, Pathway

## Abstract

**Background:**

The increased number of older adults living alone has created a substantial care need. However, the utilization rate of services and facilities to meet these needs are surprisingly low. Many of older adults experience difficulties accessing these services, although it remains unclear how these obstacles impede access to services. This study explored the obstacles and difficulties experienced by urban older adults in seeking community care.

**Methods:**

A phenomenological study was carried out and participatory observation and in-depth interviews were employed to investigate the process of seeking care of older adults in urban communities. A total of 18 urban community-dwelling older adults aged 75 years and over were included. Data collected were analysed by content analysis.

**Results:**

We identified the pathways by which senior-only households sought community care and encountered obstacles. (1) lack of community care information: older adults did not know where and how to get services, even though the care institutions scattered throughout the community; (2) limited mobility: older adults often suffered from various chronic diseases, which physically hindered their access to care resources; (3) complex process of achieving care: the functional fragmentation and geographical dispersion of care institutions made the care-seeking process challenging and confusing for older adults; (4) incomprehension of needs expression: limited interaction time and communication barriers between staff of institutions and the older adults were the final obstacle. Only by surmounting these obstacles one by one can older adults access the care resources effectively.

**Conclusions:**

When older adults in the community initiated calls for help, they encountered several obstacles. Their physiological and social disadvantages limited their ability to seek care physically. Lack of integration and clear guidance in the process of providing community care exacerbated these difficulties. Reform of care services should focus on the visibility and accessibility of services for older adults.

**Supplementary Information:**

The online version contains supplementary material available at 10.1186/s12877-022-02816-y.

## Introduction

The world population is rapidly aging, especially in China. By the end of 2020, the population aged 60 or above in China had reached 264 million, accounting for 18.7% of the total population, and people aged 65 or above were up to 190 million [1]. Older adults have various care needs, including door-to-door visiting, nursing and housework, companion to visit doctors, rehabilitation and daily shopping [2, 3]. Unfortunately, the inverted pyramid family structure and empty-nesting intergenerational living style impedes traditional home care [4]. About 55% of older adults said their care needs were not met [5]. Subsequently, the government tried to satisfy these unmet needs by constructing hospitals, home-based care service centres for older adults, and day-care centres in the community that provided medical, mental, and daily life care services, such as home visits, housework assistance, rehabilitation, and dining services [6]. The community care services covering various aspects of daily life of older adults were expected to improve quality of life of older adults and their families with less financial burden [7, 8]. Additionally, compared with traditional home care, community care services provide higher quality assistance, as they are staffed with more professional workers [9].

However, a social tracking survey of older adults in China showed that these services were inadequately utilized; home visits had the highest usage rate at 3.91% [10]. The proportion of older adults using community health services was also lower than that of national various levels of hospitals [11]. Also their likelihood of choosing these primary health care was declining, contrary to the initial objective of constructing these community care institutions [12].

Generally, factors influencing engagement of older adults in various kind of social activities could be broadly divided into two categories, environmental and individual factors [13]. Environmental and infrastructure factors refers to the existence and availability of the social activities [14]. Demographic characteristics including age, education level, and income were found to be factors, although the results have been inconsistent [13, 15]. Personal health, skills, motivations, and social networks also serve as barriers/facilitators, especially social and family networks, which are protective against later barriers in ageing [16]. Therefore, more attention should be paid to older adults living alone and in senior-only households with limited support and protection [17].

Specifically, previous studies discussed the reasons for this insufficient utilization of community services from a macro-level, involving system design, community and social care institutions’ practice [18, 19]. First, several studies showed there was a general imbalance of allocation of supporting resources for older adults in social security systems in various countries [20–23]. The incomplete coverage of endowment insurance, including service content and objects, strictly restricted the utilization of care resources for older adults [24, 25]. Funding arrangement reform for supporting resources is a common problem faced by governments worldwide [4, 26, 27]. Second, for the community care practice, the current management system has not been established perfectly. There is no effective evaluation regulation, orderly medical pattern, or comprehensive coverage of health records; thus, the community care system does not operate optimally [28, 29].

Some studies sought to determine the barriers to accessing community care resources from the perspective of older adults; for example, personal social capital influences older adults’ awareness and knowledge of care services and barriers to their use [30, 31]. The mobility of older adults might affect the probability of leaving their homes, affecting access to specific care services [32, 33]. Social demographic characteristics such as gender, education level, and income also affect the utilization of long-term care resources [34, 35].

Nevertheless, these studies are fragmented concerning the characterisation of obstacles to accessing care, even though they covered different political, economic, geographic, and personal characteristics. The reason might be that the daily life of older adults is coherent rather than fragmented in space and time; likewise, various encounters are not easily dissectible. Therefore, given the dramatic mismatch between care needs and service utilization and the current research gap, we stated the core problem as follows: why did community care institutions fall shy of expectations, and why were they shunned by older adults? What setbacks did older adults experience in the process of obtaining care services? These setbacks led to a dilemma of older adults expressing objective demand for care but being unable or unwilling to make effective use of the existing care resources in the community. To answer these questions, the present study attempted to reproduce the pathway and obstacles of seeking community care from the perspective of senior-only households. Participatory observation and in-depth interviews were used to track the trajectory of their daily life and understand how various dilemmas prevented them from seeking help.

## Methods

### Research design

A phenomenological study was carried out to describe the trajectory of older adults seeking community care [36]. We employed participatory observations and in-depth interviews with older adults in two communities in Guangzhou, Guangdong, China [37, 38]. The Consolidated Criteria for Reporting Qualitative study (COREQ) checklist was followed. See complementary file for more information.

In this study, the participatory observation allowed researchers to walk into the daily life of older adults and observe their interaction with community care institutions, providing a more thorough understanding of the research question; also, it helped access the respondents and establish a good relationship of trust, on the premise of which in-depth interviews were conducted [39]. The obstacles were categorised, and the reasons behind them were explored. The fieldwork of this study was conducted from September 2018 to October 2019.

### Setting and participants

This study was conducted in two urban communities in southern China with the support of community care institutions, including social work stations, community health service centers and community neighborhood committees. Researchers first obtained the basic demographic data of the older adults in senior-only households through these community care institutions. Then, researchers visited the older adults door to door following social workers or caregivers of the institutions. Connections were established and older adults meeting inclusion criteria were recruited into the study. The participatory observations and in-depth interviews were conducted at the older adults’ home and places of their daily activity.

Older adults aged 75 or above, living alone or in senior-only households, were recruited face-to-face by purposive sampling. Recruitment stopped when no new results emerged. A total of 18 older people were included, ranging in age from 75 to 88 years old, with an average age of 82.11 ± 2.74 years. All had one or more chronic diseases such as hypertension and arthritis. The duration of the interviews ranged from 43 to 77 min, with an average time of 61.78 ± 10.55 min. Demographic characteristics of participants were shown in Table 1.

**Table 1:** Characteristic of participants

Participant	Gender	Age	Physical condition	Interview time	Living status	History of professional occupation
P01	F	82	Fracture, osteoporosis, hypertension	77 min	Alone	Staff of a public institution
P02	F	82	Hypertension, stroke, palsy	65 min	Living with her husband	Staff of a public institution
P03	M	81	Hypertension, hyperlipidemia	68 min	Living with his wife	Staff of civil service
P04	F	85	Fracture, hypertension, loin and leg pain, poor sleep quality	45 min	Alone	Professor
P05	F	83	Terminal cancer	50 min	Alone	Individual business
P06	F	83	Arteriosclerosis, fracture, hip replacement, poor sleep quality	55 min	Alone	Staff of a research institute
P07	F	83	Poor sleep quality, frequent colds	73 min	Alone	Staff of state-owned enterprise
P08	F	88	Heart disease, atherosclerosis, osteoporosis, knee pain	52 min	Alone	Staff of civil service
P09	F	83	Coronary heart disease, facial neuritis, knee arthritis, cervical spondylitis, scapulohumeral periarthritis	58 min	Alone	Staff of state-owned enterprise
P10	F	81	Arthritis, limb pain, indigestion, bone tuberculosis, poor sleep quality	43 min	Alone	Professor
p11	F	79	Moderate paralysis after stroke	52 min	Living with her husband	Professor
P12	M	82	Arthritis, hypertension, limb weakness, joint replacement	70 min	Alone	Professor
P13	F	80	Hypertension, diabetes	75 min	Living with her husband	Staff of state-owned enterprise
P14	M	75	Hypertension, arthritis	77 min	Living with his father	Staff of state-owned enterprise
P15	F	83	Arthritis, poor sleep quality	60 min	Alone	Staff of state-owned enterprise
P16	F	85	Fracture, poor vision, hypertension	55 min	Alone	Staff of a research institute
P17	M	83	Hypertension, parkinsonism	72 min	Alone	Staff of a college
P18	M	80	Hypertension	65 min	Alone	Staff of a public institution

*Abbreviations: P* participant, *M* male, *F* female

### Data collection

Participatory observation and in-depth interviews were used to collect data. After recruitment and obtaining their consent, researchers lived with the older adults for two or three days, to observe but not interfere in their daily life, including everyday activities, going out and social interactions. Notes were taken whenever possible and conversations between the older adults and researchers or community care institutions workers were recorded. Process of participatory observations allowed researchers to build a trusting relationship with the participants, on which in-depth interviews were conducted. Five researchers conducted the observations and interviews in total, while every visit was made by two researchers or a social worker/caregiver and a researcher.

Interview guide was developed based on a literature review and revised by a gerontologic nursing specialist, a nursing anthropology specialist, and a social worker [40, 41]. The final interview questions focused on daily diet, activity, medical care for acute and chronic diseases, community care services, and obstacles to access. Examples of questions are as follows: Have you had any problems in your life recently? (Prompts: How do you have meals usually? Do you have anyone to talk to? How do you solve the problem of seeing a doctor?) Do you know what care services are provided for you by the Community Neighbourhood Committee, Social Work Station, and Home Care Service Centre? (Prompts: Have you ever been to a community canteen for the elderly? Have you ever been visited by the neighbourhood committee or home care centre?) Why didn’t you join a community interest class? (Prompts: Never heard of it? Or is it something else?).

The interviews were conducted when the time was appropriate for the participants and live recordings were conducted after informed consent was obtained. Prior to the semi-structured interviews, researchers chatted about daily routine and physical conditions with participants. Since the interview topic was about the experience of seeking community care in daily life, it was easy and natural to turn to the interview questions.

### Data analysis

Data collected by participatory observation and in-depth interviews were analysed by qualitative content analysis [42, 43]. The interview and reflective dialogue recordings were transcribed verbatim by one researcher within 24 h and checked sentence-by-sentence by another to ensure accuracy.

Researchers read the transcripts several times to obtain the sense of whole. After dividing into two groups (group 1: NG, XW and MZ; group 2: YM, QD and WZ), researchers independently identified and condensed the meaning units in the text; the condensed meaning units were abstracted and labelled as codes; the various codes were sorted into sub-categories and categories based on their differences and similarities [44]. Then the discrepancies between the two sets of categories were compared and discussed in the whole research team, including a nursing professor, two medical anthropologists and several nursing students, until consensus on how to sort the codes was reached. Finally, potential themes of broader significance were formulated from the generated categories. The above preparation, coding, creating categories and abstraction steps were repeated until no further themes could be identified [44]. NVivo 11 was used to manage the content analysis.

Considering the respondents were of an older age, we confirmed their responses repeatedly during interviews. The transcripts and themes extracted from the analysis were also confirmed with the older adults to guarantee the trustworthiness and rigor of the study.

### Ethical considerations

The Ethics Committee of the School of Sociology and Anthropology, Sun Yat-sen University, approved the study (IRB No. SYSUIBRDA20180816), which was conducted in strict compliance with the Declaration of Helsinki (WMA). The study was carried out with the support of the local social work stations, health service centres, and neighbourhood committees. Every door-to-door visit was made by at least two persons, including a social worker/caregiver and a researcher or two researchers, to deal with safeguarding issues. Before conducting participatory observations and in-depth interviews, all participants were informed of the purpose and content of the study and provided written informed consent. Participants understood that they could discontinue the study at any time. All data were used for research purposes only and were not accessible to anyone outside the research team.

## Results

**Fig. 1:**
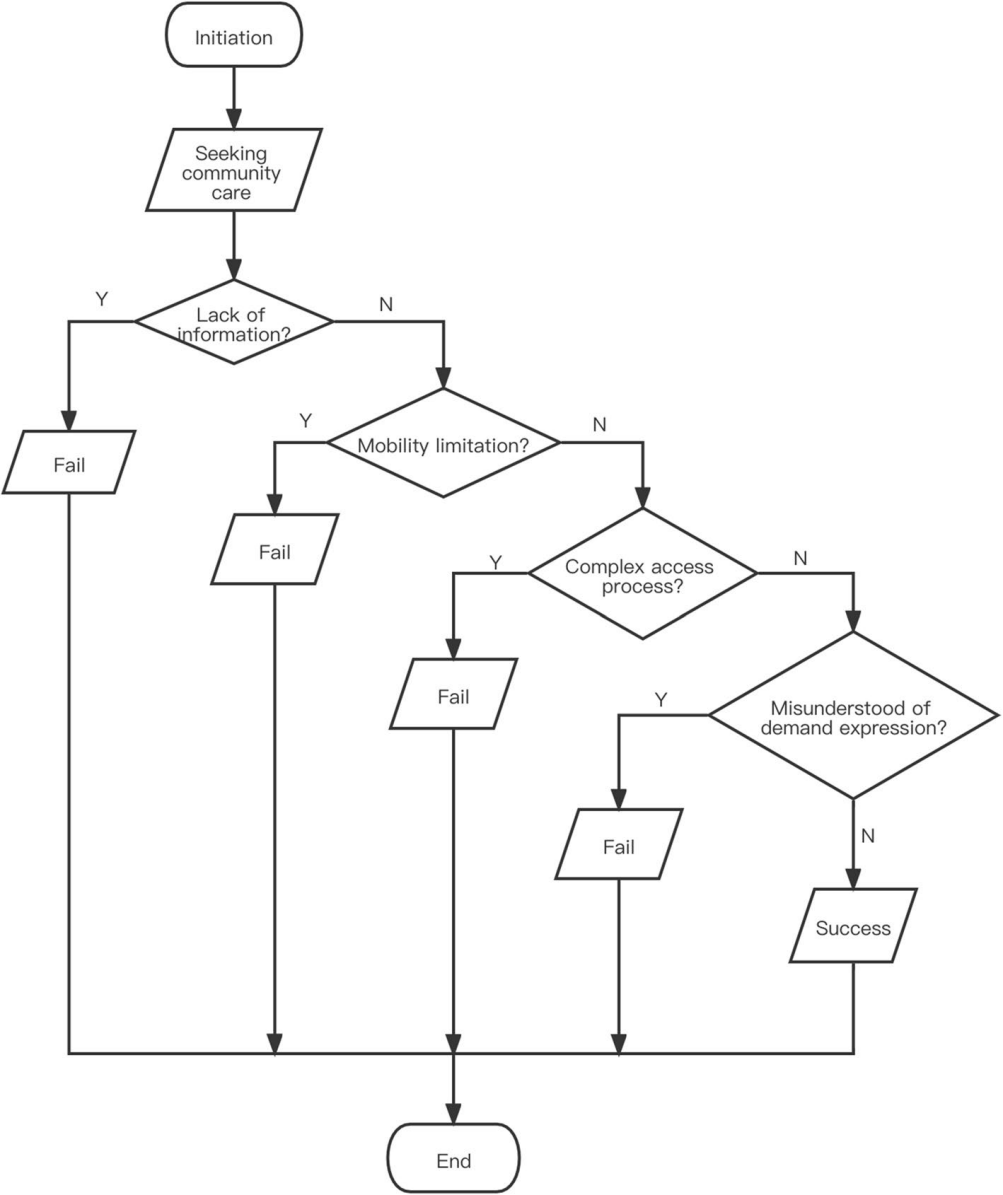
Analysis of the access path of community care for urban senior-only households

The process of seeking care services was a journey characterised by many difficulties for the older adults in urban communities. They endured many hardships with frail bodies to satisfy their needs. We presented these obstacles as they experienced them in the real world. Being aware of surrounding care services is a prerequisite. While obtaining appropriate care services, older adults overcame the obstacles of lack of information, mobility limitation, complex access process, and misunderstanding of demand expression (Fig. 1). ‘Fail’ in the figure refers to not accessing care and ‘success’ refers to accessing care. Each of these obstacles was likely to lead to failure in seeking care, and all of them must be overcome to obtain care successfully.

### Lack of care information

Awareness of nearby care resources is the basis of access to care. Although the care institutions existed, scattered throughout the community, they were invisible and unreachable for older adults, confined to small spaces with relatively isolated information sources. Three participants indicated they ‘never heard of social work stations or home endowment service centres’ or unified the organisations such as ‘Neighbourhood Party Committee’ or ‘Street Party Committee’. Ambiguous designations reflected their unfamiliarity with community care services.

‘I dare not go to the hospital alone, I want someone to take me to cure my arthritis, but I don't know who to turn to.’ *(P15)*

An 80-year-old mother-in-law also expressed this dilemma. She had sought help to get her husband to a doctor; however, she had no idea of the existence of a community home assisted-living centre across the street, let alone its ‘accompanying medical’ programme. After trying everything in vain, she finally gave up taking her husband to see a doctor.

‘My husband is getting old, I can't take him by myself, I don't have my children around, and they’re busy with work. I really don't know who to turn to for help, so I can only give up.’ *(P13)*

We found that older adults’ most common complaints were that they had no access to help for medical care needs; the lack of information about the source of care even led to medical accidents. A participant, independently looking after his 95-year-old father who was paralysed and bedridden; however, the weekly replacement of a bladder catheter became a challenge.

‘My father's catheter should be changed once a week, but because of my advanced age, I am powerless to take him to the hospital. The taxi is also reluctant to pick up an old man with urinary incontinence. Currently, the change period had to extend to a month. We know that the risk of infection is very high, and we are looking for a visiting service to replace the catheter, but we don't know where to look for.’ *(P14)*

The trend towards the electronisation of community care services in the information age has also become a barrier for older adults. The communities in this study had launched online help services. Although social workers had personally come to teach older adults how to use smartphones, some old people still did not know how to operate them.

‘I used to be taught how to use a mobile phone, but I'm too old to learn. I still don't know what to do when an emergency happens.’ *(P05)*

Lack of care-related information was the first obstacle to older adults seeking services. If older adults do not know where to obtain help, they cannot know of community care facilities or how to use them. As a result, the care-seeking process terminates, and care access fails.

### Restricted mobility

After learning where and how to use services provided by the community, older adults living alone in urban communities access care services on their own. However, physical mobility determines whether they could access medical and routine care. Mobility constraints were the second barrier to access to care resources.

Chronic diseases and other comorbidities often accompany advanced age. All participants in the study had at least one chronic disease, including osteoporosis, arthritis, or hypertension. Fractures, hip and knee arthritis and other diseases that severely limit activity occurred at 66.7%. Movement difficulties were among the common complaints.

‘I am so weak that I can’t go to the market from home. The feeling is terrible.’ *(P17)*

‘My knee osteoarthritis is quite serious, and I can't bend it completely, so I can't mop the floor for cleaning. I'm worried about falling.’ *(P09)*

These mobility difficulties, which were initially motivations for older adults to seek care, dramatically become obstacles to their access to care in turn. One of our participants, who underwent hip replacement surgery in 2017, had to drag her frail body to go out alone to see a doctor every two weeks.

‘I fell in the elevator earlier, and after that, it became more difficult to move. I won't go out unless I really need it. But I still have to go out to see a doctor. I am afraid to slip on the moss near the door. When I was younger, the journey would take 10 minutes. Now, it takes 20 or 30 minutes.’ *(P06)*

A grandmother used to take meals in the community ‘canteen for the elderly’ (a canteen designed for older adults to provide healthy and nutritious meals; prices are subsidised according to age); however, since suffering a broken leg, she could not enjoy the convenience of the canteen and needed to buy food and cook by herself.

‘Originally, I go to the canteen for the elderly to have lunch and buy a little more for dinner. But since I can’t walk because of the fall, I have to cook by myself.’ *(P16)*

A 85-year-old grandmother was often involved in community activities when she was young, but currently she became unable to go outside because of unsteadiness on her feet.

‘The only way to keep up with time is to get out there, to meet people, to say hello and ask people what's new; otherwise, you wouldn't know anything. I used to go to community retirement meetings, but now I don't go because I'm physically handicapped, and my social circle has narrowed to one.’ *(P04)*

The prevalence of chronic diseases such as osteoporosis and joint damage limits mobility and makes it impossible for older adults to seek care resources. However, such a problematic path did not end with a single clear destination; the scattered distribution of various functional institutions in the community was another challenge.

### Complex process of care acquisition

For older adults, seeking services was a process of breaking integrative daily life into discrete demands, corresponding to different functional institutions in the community. The division of labour in these institutions was differentiated but overlapping, which were challenging as older adults frequently confused the terms ‘neighbourhood’, ‘social work station’ and ‘home care service centre’.

‘I called the neighbourhood committee to find someone to accompany me to see a doctor, but they ask me to go to the social work station to make an appointment in advance; it's really troublesome.’ *(P01)*

‘Older people, like my father, who can't take care of himself can apply for care subsidies, but to apply for the subsidy, we need a 'useless' proof issued by the hospital. But the hospital didn’t give us the certificate; then, the community did not grant my allowance. They (referring to the community workers) also knew our situation but still asked for proof. It’s too complex; I don't know how to do it now.’ *(P14)*

‘I want to find someone to help me clean my home. I heard that the home care centre can come to help doing housework, but they said they need to come to assess my situation, then go up to the application, and then to approval. The whole process takes more than 20 days, so it's too long.’ *(P06)*

The fragmentation of these institutions in physical space also complicated access to care.

As early as the ‘Community Home Elderly Meals and Matching Project’ launch, one of the grandmothers had already learned about catering service standards and applied for them. However, she had to cross an overpass to reach the dining hall. The dozens of pedestrian passage stairs were challenging for an old lady whose knees could not bend completely.

‘In the evening, there is a meal for the elderly, but I have to bypass the footbridge next to Wushan Road to get to the restaurant of the Chinese Normal University. It's a long way and a lot of trouble, so I don't go now.’ *(P09)*

When complex institutional settings collide with fragmented physical space, the acquisition of care services for older adults is a problem that consumes substantial time and energy.

‘I heard there’s an application on mobile phones that monitors the heart rate and calls for help automatically in an emergency. I went to the neighbourhood committees to consult; the workers said it’s not their job and asked me to go to the home care service centre. Then I went there and found that the materials were incomplete. It took me several trips back and forth to finally get it right. You know, my ability to walk is not very convenient. I wouldn’t have done it if I wasn't worried that no one would help me when something urgent happened.’ *(P10)*

Dispersed functional institutions are negative experiences for older adults. Sometimes they must give up seeking care services.

### Misunderstanding of requirements

When older adults finally contact the staff of care institutions after overcoming the obstacle of lack of information, limited mobility, complicated access to care, and the divergence between expression and understanding among parties become the final obstacle to accessing care.

#### Limited interaction time

To implement the appropriate provision of care services, community workers need to evaluate the self-care ability of each older adult. With a ratio of 1 to 6000 social workers to older adults, the actual assessment time for each older adult is limited to only 15 min. A 15-min interview is often insufficient to provide a holistic and accurate assessment of their real and urgent needs and older adults expressing unmet care needs.

‘These people always came and talked for only ten minutes. We didn’t have a good chat, and they left quickly. Some words I didn’t even understand; also, I don’t know whether I expressed myself clearly.’ *(P03)*

‘There used to be volunteers visiting me before. All of them were very nice, talking with me and buying food for me. But no one has come for a long time. You (the researcher) must help me to report the phenomenon. I really wish someone would come to chat with me.’ *(P07)*

#### Communication barriers

Communication barriers derived from differences between Mandarin and other dialects (Cantonese, a dialect widely used in central and western Guangdong, southeast Guangxi, Hong Kong, Macao, and some countries or regions in Southeast Asia and overseas Chinese communities).

Some local older adults in Guangzhou could only understand Cantonese; however, the staff or volunteers in social work stations and other institutions might not speak Cantonese. Hence some of the local residents complained, ‘I can’t understand Mandarin, don’t you have Cantonese speakers to communicate with me? ’ *(P12).*

As professionals, community social workers use a set of professional terms exclusive to the service system, and they used these terms intentionally or unintentionally when communicating with older adults. For example, when older adults knew the telephone number of the social work station and tried to contact the workers, older adults individual was referred to as a potential case object, which needed to be further confirmed as a consultation case and followed up. The technical terms like ‘individual case’ were incomprehensible for older adults.

‘I was alone and unattended at home. I am so lonely in my 80s. When asking the street (Street office, the administrative institution of the town administrative district in China), I didn't understand what they were saying.’ *(P07)*

‘Limited interaction time’, ‘communication barriers’, any kind of poor communication with the staff of the care agency could cause the care-seeking journey to fail. Only after surmounting these obstacles one by one, care resources might be genuinely provided to older adults.

## Discussion

This study described the paths and obstacles of urban senior-only households concerning community care services. We found that when older adults in the community initiated calls for help, they encountered resistance before eventually obtaining help from community. In this process, knowing where and how to seek help were prerequisites to obtaining care. Instead of an unimpeded path, older adults struggled to navigate a complicated administrative system in a complex urban space.

Older individuals’ social circles shrink with age. In China, almost half of the urban older adults, even those with relatively superior education and economic conditions, did not participate in any social activities (i.e., community entertainment, group sports, internet surfing, and others) [45]. The incidence of social isolation, defined as ‘lack of belonging, social interaction and enriching high-quality social relationships’, reached 43% [46]. This unsatisfactory social state did not provide adequate social support for older adults, such that they were exposed to an increased risk of cognitive impairment, which further worsened their declines [47]. At present, community services are being updated and developing towards intelligent electronic networks [48]. Although service procedures have been greatly simplified, they remain challenging for older adults to understand and implement [49]. Despite the demand, the lack of relevant knowledge hinders service utilization, and this may be a fundamental reason why the demand for care far exceeds the low service utilization rate [50]. The problem of lack of knowledge about community care services also appeared in some developed countries [19]. A study from Norway found that some residents did not know where to seek community care for older adults with dementia, hindering the use of care services [51]. In the present study, 3/18 of older adults could not distinguish between the neighbourhood committee, social work station, home care service centre, and other departments. It was difficult to determine the actual functions of the various departments; thus, the individuals lose the possibility of obtaining care.

Second, older adults often suffer from chronic diseases, with 75% of those aged 60 or above suffering from at least one chronic disease [52]. These diseases severely affected their quality of life and limited mobility, making it impossible to scramble to satisfy a single need while incurring additional unmet needs [53]. These findings suggest that older adults are at a disadvantage because of a lack of necessary strength and skills.

At the same time, lack of integration and clear path guidance in the process of community care delivery exacerbated the difficulties of seeking care services for older adults [54]. On the one hand, there was a lack of integration of community services, different affairs were subordinate to different departments and agencies [19, 55, 56]. Thus several transactions were likely to be duplicated or contradictory because of a lack of coordination among departments; such fragmentation may frustrate older adults, increasing the uncertainty of demand realisation [57]. A recent review of geriatric health care systems in Asia found that decentralisation of health services hindered the implementation of comprehensive and continuous care and emphasized the integration of health care services, where doctors provided health services with the related health services to meet the various health needs of older adults [58]. Since activities of daily living comprise many aspects, a series of care services are needed to support independent living.

On the other hand, the spatial distribution of community service agencies was overly scattered and was not geographical accessible for older adults with chronic diseases and mobility difficulties [59]. Spatial accessibility has been emphasized in previous studies [60, 61]. Unfortunately, the functional fragmentation and geographical dispersion of care institutions often coexist in the real world [62]. As a result, older people often encounter snags at every turn. This situation occurs because independent administrative operations are relatively convenient for each department to manage separately [56]. Thus, the institutions bearing the care functions are separated and scattered, despite making the process of seeking care for older adults complex and unworkable. In short, the current model of care services is a top-down management model; while the process of acquiring care resources upward requires constant trial and error; it is simply too difficult for older adults who suffer various inconveniencies to navigate the system [63]. On the basis of the reality, some countries like Korea have tried to integrate local services and resources and develop a community-based integrated service model to help older adults in “aging in place” [64].

Our findings highlight the need to inspect the care-seeking process from the perspective of older adults. Systems worldwide are fortifying care support for older adults; however, the actual utilization rate remains unsatisfactory. Only by reconstructing real-life care-seeking paths could we systematically and comprehensively dissect the obstacles to accessing objectively sufficient resources. Every step of older adults’ quest for care was fraught with challenges. Any insurmountable obstacle would terminate the quest for care, and multiple dilemmas often existed simultaneously. The disadvantages of older adults and the inconveniences in the care mechanism were the reasons for these obstacles. Fundamentally, the current care model, which was easy to manage from top to bottom, was not conducive to successful access to care services from bottom to top. Therefore, to develop an aged-friendly service system, we must take older adults’ experience into consideration and make the access to services unimpeded. In conclusion, for the provision of care and services for older adults, the focus should not be solely on the construction of basic facilities, the layout of institutions, and the provision of specific services. Instead, it should focus on the accessibility and visibility of services.

## Strengths and limitation

The primary strength of the study was the systematic identification of the obstacles that the older adults faced in seeking community care. The participatory observation and in-depth interviews helped researchers build relationships with older adults, present their real life and interactions with community care institutions in great detail and show how various obstacles effectively prevent them from seeking care.

The main limitation was that the study focused on the senior-only households in urban communities in relatively developed areas of southern China and did not involve the current care needs and services utilization of older adults living in rural and less developed areas. The process of seeking care and obstacles faced by those may be different.

## Conclusion

We reconstructed the paths and obstacles encountered by senior-only households in seeking community care services. Understanding information was the premise for older adults to initiate their help-seeking behaviour. However, they were limited by poor understanding of information sources and limited learning ability; the ever-changing care system also increased difficulties of accessing information. Even if they knew how to get help, their limited mobility prevented them from moving around in pursuit of a demand, thus they had to forgo opportunities to enjoy resources. The design of the community care system appeared to be not conducive to access to services for vulnerable older people. They had to face complicated procedures in limited interaction times. The difficulty of older adults seeking community care service could be attributed to the top-down management of the current care model.

### Implication and future research

The various obstacles encountered by older adults demonstrated the hardship in seeking community care. That may be the reason why older adults with objective demand for care were unable or unwilling to make effective use of the existing care resources in the community. Therefore, the reform of long-term care service should pay attention to the visibility and accessibility of the service from the perspective of older adults. Since the lack of integration of care services and geographical inaccessibility exacerbated difficulties in seeking care, the current care institutions should be more integrated in service contents and spatial settings.

The present study abstracted the various obstacles into a process which covered the common features of obstacles experienced by older adults with different conditions in seeking care. We also appeal for future research to include more samples and compare the different obstacles encountered by different older adults to present the process in more perspectives. Furthermore, since even older adults in urban communities are still faced with various difficulties when seeking care, those with more scarce care resources in rural and less developed areas may experience even greater difficulties, and thus deserve more attention.

## Supplementary Information

**Additional file 1.**

## Data Availability

The datasets generated and analyzed during the study are available from the corresponding author by reasonable request.
