# Supplementary material for: Obstacles to access to community care in urban senior-only households: a qualitative study
Source: BMC Geriatr. 2022 Feb 14;22:122. doi: 10.1186/s12877-022-02816-y (PMC8842867; doi:10.1186/s12877-022-02816-y)
Supplement: Supplementary file 1 — Additional file 1. [file 12877_2022_2816_MOESM1_ESM.docx]

**Reporting Guideline Checklist**

**（Consolidated criteria for reporting qualitative studies, COREQ）**

| **No Item** | **Guide questions/description** |  |
| --- | --- | --- |
| **Domain 1: Research team and reflexivity**  **Personal Characteristics** | |  |
| 1. Interviewer/facilitator | Which author/s conducted the interview or focus group? | Ni Gong, Ya Meng, Qian Hu and Qianqian Du |
| 2. Credentials | What were the researcher’s credentials? E.g. PhD, MD | Ni Gong- PhD  Ya Meng- Postgraduate student  Qin Hu- Postgraduate student  Qianqian Du- Postgraduate student  Xiaoyu Wu- Postgraduate student  Wenjie Zou- Postgraduate student  Mengyao Zhu- Postgraduate student  Jiayan Chen- BD  Lan Luo- BD  Yu Cheng- PhD  Meifen Zhang- PhD |
| 3. Occupation | What was their occupation at the time of the study? | Ni Gong- Associate professor  Ya Meng- Postgraduate student  Qin Hu- Postgraduate student  Qianqian Du- Postgraduate student  Xiaoyu Wu- Postgraduate student  Wenjie Zou- Postgraduate student  Mengyao Zhu- Postgraduate student  Jiayan Chen- Social worker  Lan Luo- Nurse  Yu Cheng- Professor  Meifen Zhang- Professor |
| 4. Gender | Was the researcher male or female? | Ni Gong- Male  Ya Meng- Female  Qin Hu- Female  Qianqian Du-Female  Xiaoyu Wu-Female  Wenjie Zou- Female  Mengyao Zhu- Female  Jiayan Chen- Female  Lan Luo- Female  Yu Cheng- Male  Meifen Zhang-Female |
| 5. Experience and training | What experience or training did the researcher have? | Ni Gong- Completed a large number of qualitative studies  Ya Meng- Community nursing internship experience  Qin Hu- Community nursing internship experience; Qualitative research article writing experience  Qianqian Du- Community nursing internship experience; Qualitative research article writing experience  Xiaoyu Wu- Community nursing internship experience  Wenjie Zou- Community nursing internship experience  Mengyao Zhu- Community nursing internship experience  Jiayan Chen- Community nursing  Lan Luo- Community nursing  Meifen Zhang- Rich experience in geriatric nursing research  Yu Cheng- Rich experience in anthropological study |
| **Relationship with participants** | |  |
| 6. Relationship established | Was a relationship established prior to study commencement? | We didn’t establish a relationship with participants prior to study commencement. |
| 7. Participant knowledge of the interviewer | What did the participants know about the researcher? e.g. personal goals, reasons for doing the research | The participants know about reasons for doing the research，and the purpose of the research. |
| 8. Interviewer characteristics | What characteristics were reported about the interviewer/facilitator? e.g. Bias, assumptions, reasons and interests in the research topic | The interviewers want to explore obstacles and difficulties experienced by urban elderly individuals seeking community care |
| **Domain 2: study design** | | |
| **Theoretical framework** | | |
| 9. Methodological orientation and Theory | What methodological orientation was stated to underpin the study? e.g. grounded theory, discourse analysis, ethnography, phenomenology, content analysis | Phenomenology |
| **Participant selection** |  |  |
| 10. Sampling | How were participants selected? e.g. purposive, convenience, consecutive, snowball | Purposive sampling |
| 11. Method of approach | How were participants approached? e.g. face-to-face, telephone, mail, email | Face-to-face |
| 12. Sample size | How many participants were in the study? | 18 |
| 13. Non-participation | How many people refused to participate or dropped out? Reasons? | None |
| **Setting** |  |  |
| 14. Setting of data collection | Where was the data collected? e.g. home, clinic, workplace | The elderly’s home, community social work station, neighbourhood committee |
| 15. Presence of non-participants | Was anyone else present besides the participants and researchers? | Sometimes there would be social workers or care institutions staff. |
| 16. Description of sample | What are the important characteristics of the sample? e.g. demographic data, date | The urban elderly living alone or in senior-only households. |
| **Data collection** |  |  |
| 17. Interview guide | Were questions, prompts, guides provided by the authors? Was it pilot tested? | Yes |
| 18. Repeat interviews | Were repeat interviews carried out? If yes, how many? | No |
| 19. Audio/visual recording | Did the research use audio or visual recording to collect the data? | The research used audio recording to collect the data. |
| 20. Field notes | Were field notes made during and/or after the interview or focus group? | Field notes made during the interview |
| 21. Duration | What was the duration of the interviews or focus group? | From 43 to 77 minutes |
| 22. Data saturation | Was data saturation discussed? | Yes |
| 23. Transcripts returned | Were transcripts returned to participants for comment and/or correction? | Yes |
| **Domain 3: analysis and findings** | |  |
| **Data analysis** | |  |
| 24. Number of data coders | How many data coders coded the data? | Six |
| 25. Description of the coding tree | Did authors provide a description of the coding tree? | No |
| 26. Derivation of themes | Were themes identified in advance or derived from the data? | Derived from the data |
| 27. Software | What software, if applicable, was used to manage the data? | NVivo 11 |
| 28. Participant checking | Did participants provide feedback on the findings? | Yes |
| **Reporting** | |  |
| 29. Quotations presented | Were participant quotations presented to illustrate the themes / findings? Was each quotation identified? e.g. participant number | Yes |
| 30. Data and findings consistent | Was there consistency between the data presented and the findings? | Yes |
| 31. Clarity of major themes | Were major themes clearly presented in the findings? | Yes |
| 32. Clarity of minor themes | Is there a description of diverse cases or discussion of minor themes? | Yes |
